# Autophagy regulates death of retinal pigment epithelium cells in age-related macular degeneration

**DOI:** 10.1007/s10565-016-9371-8

**Published:** 2016-11-29

**Authors:** Kai Kaarniranta, Paulina Tokarz, Ali Koskela, Jussi Paterno, Janusz Blasiak

**Affiliations:** 1 Department of Ophthalmology, University of Eastern Finland, 70210 Kuopio, Finland; 2 Department of Ophthalmology, Kuopio University Hospital, 70210 Kuopio, Finland; 3 Department of Molecular Genetics, University of Lodz, 90-236 Lodz, Poland

**Keywords:** Age-related macular degeneration, Cell death, Apoptosis, Necroptosis, Pyroptosis, Alu transcripts

## Abstract

Age-related macular degeneration (AMD) is an eye disease underlined by the degradation of retinal pigment epithelium (RPE) cells, photoreceptors, and choriocapillares, but the exact mechanism of cell death in AMD is not completely clear. This mechanism is important for prevention of and therapeutic intervention in AMD, which is a hardly curable disease. Present reports suggest that both apoptosis and pyroptosis (cell death dependent on caspase-1) as well as necroptosis (regulated necrosis dependent on the proteins RIPK3 and MLKL, caspase-independent) can be involved in the AMD-related death of RPE cells. Autophagy, a cellular clearing system, plays an important role in AMD pathogenesis, and this role is closely associated with the activation of the NLRP3 inflammasome, a central event for advanced AMD. Autophagy can play a role in apoptosis, pyroptosis, and necroptosis, but its contribution to AMD-specific cell death is not completely clear. Autophagy can be involved in the regulation of proteins important for cellular antioxidative defense, including Nrf2, which can interact with p62/SQSTM, a protein essential for autophagy. As oxidative stress is implicated in AMD pathogenesis, autophagy can contribute to this disease by deregulation of cellular defense against the stress. However, these and other interactions do not explain the mechanisms of RPE cell death in AMD. In this review, we present basic mechanisms of autophagy and its involvement in AMD pathogenesis and try to show a regulatory role of autophagy in RPE cell death. This can result in considering the genes and proteins of autophagy as molecular targets in AMD prevention and therapy.

## Introduction

**Fig. 1:**
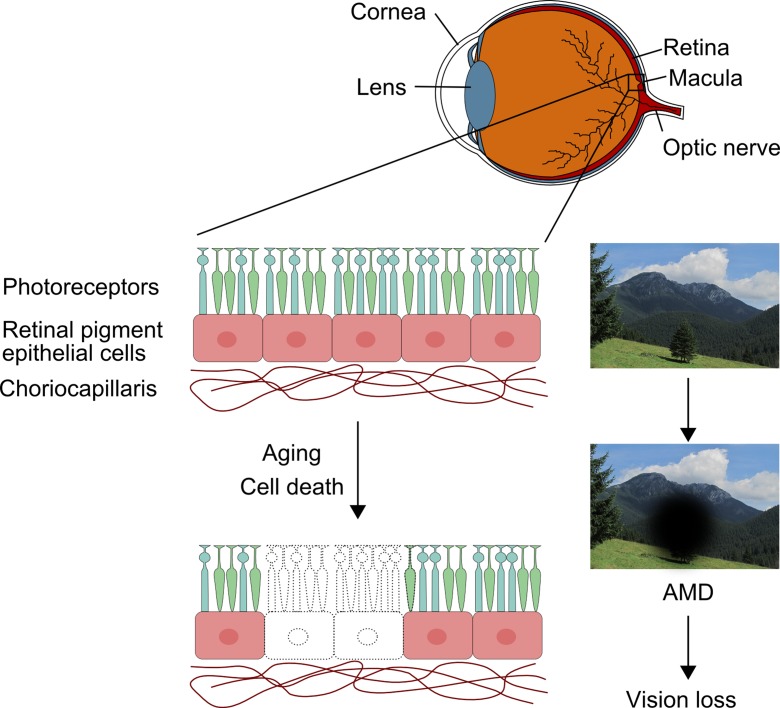
Age-related macular degeneration (AMD) affects the macula, a specific part of the central retina. It involves extensive death of retinal pigment epithelium cells, photoreceptors, and choriocapillaris and leads to a progressive loss of central vision and eventually blindness

Autophagy is an important process resulting in lysosomal degradation of unused and damaged cellular components, which can produce energy and nutrition necessary for the cell homeostasis and functions (reviewed in Mizushima and Komatsu (2011)). Autophagy plays a role in the pathogenesis of many human diseases, including cancer, diabetes, neurodegenerative disorders, and infectious diseases (reviewed in Jing and Lim (2012)). These include age-related macular degeneration (AMD), an eye disease, which is a major reason of blindness in the elderly in developed countries (Ferrington et al. 2016; Reibaldi et al. 2016). It is a complex disease occurring in its advanced stage in two major forms: dry and wet. Unfortunately, there is no effective treatment targeting both AMD forms. As AMD is a degenerative disease, its expression is associated with the degeneration of important functional parts of the retina, resulting from the death of retinal pigment epithelium (RPE) cells, photoreceptors, and choriocapillaris (Fig. 1).

Various cell death modes are considered to play a role in AMD, including apoptosis, pyroptosis (cell death dependent on caspase-1), and necroptosis (regulated necrosis dependent on the proteins RIPK3 and MLKL, independent of caspases). However, there are some conflicting reports on the regulation of the choice of a particular cell death type in AMD, but surely, cells do not simply “choose” a certain death pathway, as all different pathways influence each other in a molecular crosstalk. Aberrant autophagy has been mentioned frequently to associate with AMD, and autophagy itself is considered a kind of programmed cell death, so it can play a role in cell death in AMD (Shimizu et al. 2010) (reviewed in Tsujimoto and Shimizu (2005)). In addition, apoptotic signaling can directly regulate autophagy and vice versa (reviewed in Yonekawa and Thorburn (2013)). In general, aberrant regulation of programmed cell death by autophagy is linked with many human diseases (reviewed in Mizumura et al. (2014a)) (Mizumura et al. 2014b). Therefore, it seems reasonable and justified to study the role of autophagy in the regulation of RPE cell death in AMD.

## Age-related macular degeneration—an eye disease involving extensive cell death

Age-related macular degeneration is an eye disease affecting mainly elderly people, and it is a major cause of blindness among individuals over 65 years in developed countries (Reibaldi et al. 2016). As mentioned, two basic forms of advanced AMD are dry (non-exudative) and wet (exudative), but early dry AMD is hardly detectable, and its advanced form, called geographic atrophy (GA), which is associated with a massive loss of retinal cells, is responsible for a substantial part of blindness related to AMD (Klein et al. 2007).

AMD is a complex disease with genetic and environmental factors involved in its etiology. Although the exact mechanism of its pathogenesis is not known, oxidative stress and inflammation can play a role (reviewed in Kauppinen et al. (2016)). However, it is not exactly known how oxidative stress can contribute to the degeneration of RPE cells, and two mechanisms of their death, apoptosis and necrosis, are considered and supported by both in vivo and in vitro studies. Apoptosis is frequently attributed to RPE cell death in AMD, and both the caspase-dependent and -independent pathways are suggested to be involved (reviewed in Adler et al. (1999), Ferrington et al. (2016), and Hanus et al. (2015a)) (Dunaief et al. 2002; Hanus et al. 2015b; Rodrigues et al. 2011; Tsao et al. 2006; Wang et al. 2012). Hanus et al. (2013) suggested that necrosis, and not apoptosis, is a dominant cell death in RPE cells subjected to oxidative stress. In that study, the contribution of autophagy to RPE cell death was not observed. We also noted a dominancy of necrotic cell death in ARPE-19 cells treated with *tert*-butyl hydroxyperoxide (Tokarz et al. 2016). However, the problem of the involvement of necrosis in RPE cell death is still controversial and needs further studies.

Several issues should be taken into account in considering mechanisms of cellular death in AMD, among them two seem to be especially important. Firstly, AMD is a disease, which phenotype can dramatically change with its progression. Secondly, the region of the retina affected in AMD contains a highly heterogeneous population of cells, including RPE cells, choriocapillares, and neurons. The survival of neurons in the retina is regulated in a much more complex way than that of RPE cells, as these post-mitotic cells must function within neuronal circuits in the organism (reviewed in Kole et al. (2013)). Many factors can contribute to survival and death of retinal cells, but some of them can act differently in different cells, depending on their receptors (reviewed in Adler et al. (1999)). This mechanism is even more complicated by the concept that AMD can be associated with disturbed cell communication (reviewed in Nakahara et al. (2013)). This effect can result in a receiving of a death signal by one type of cells and transmitting it, in a changed form, to other types. Alternatively, cells receiving this primary signal can change the local environment, which affects other cells leading to their degeneration. Recruitment of inflammatory cells, changed cellular phenotypes, and autocrine and paracrine signaling alterations certainly modify the local tissue environment that may finally lead to cell death.

## Autophagy—at the crossroad between cell death and survival

**Fig. 2:**
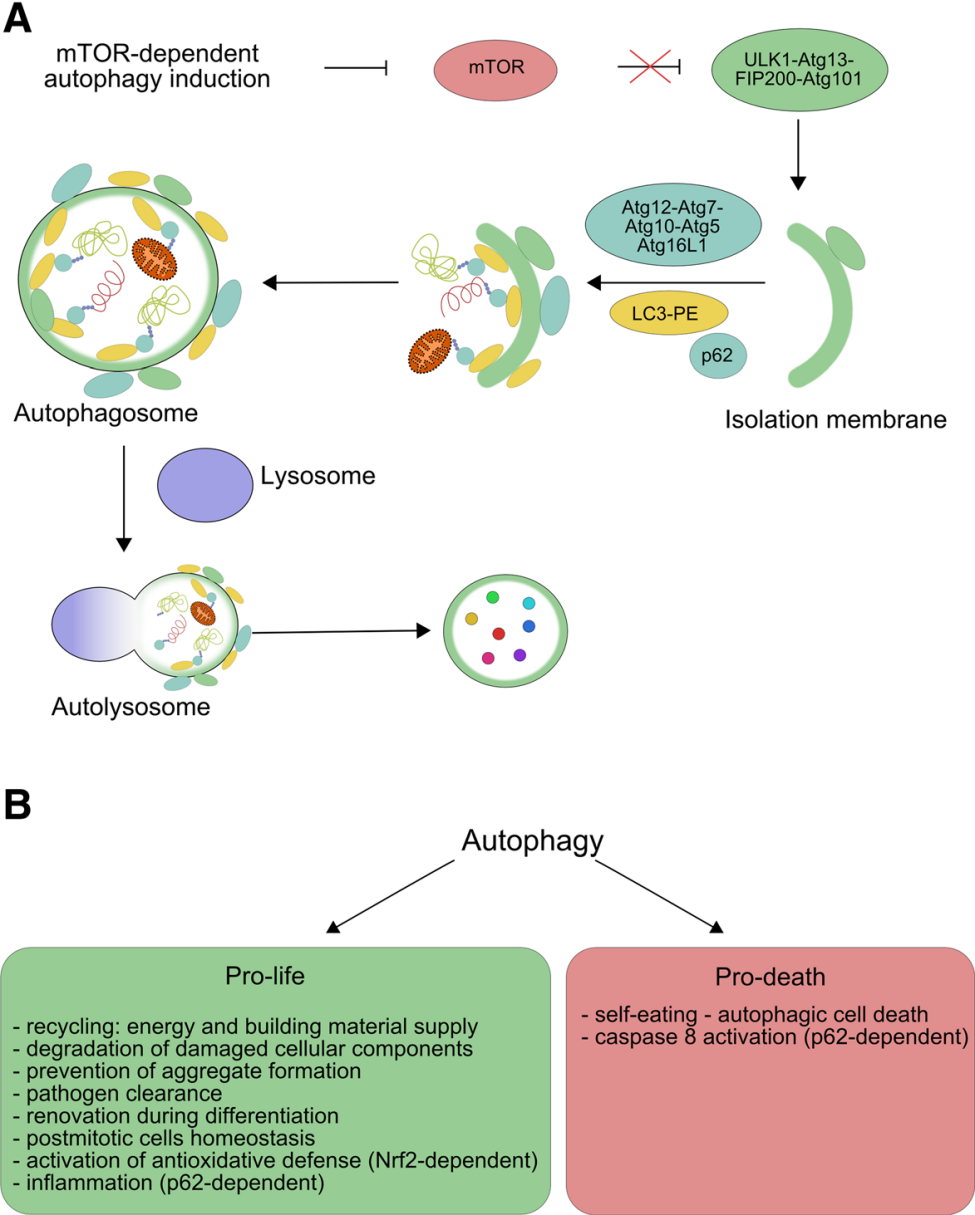
Damaged and unneeded proteins and organelles as well as other cellular component-derived objects (cargo) are enclosed by an isolation membrane to form an autophagosome, which combines with the lysosome forming an autolysosome, where the cargo is degraded by a concerted action of lysosomal enzymes and autophagy-related proteins (Atgs) giving various products, including amino acids, which can be used by the cell (*small circles*). Many proteins are involved in this process, but in mammalian target of rapamycin ( mTOR)-dependent autophagy, the key event in the formation of the autophagosome is inactivation of mTOR, resulting in recruitment of the FIP200 and many Atg proteins. Microtubule-associated protein 1A/1B-light chain 3 (LC3) is conjugated with phosphatidylethanolamine (PE) to bind autophagosome membrane, but then, it is degraded (**a**). Autophagy plays many important functions in the cell, which can be divided into two classes, pro-life and pro-death, which are associated with coping with unfavorable extra- and intracellular environment and functions contributing to elimination of faulty cells (**b**)

Cytosolic components, including organelles, undergo changes, which can result in their uselessness and should be eliminated from, or better, being recycled in the animal cell. This is important also for providing building block to assemble needed biomolecules and fuel for energy-cost metabolic processes. Autophagy, sometimes called self-eating, directs degradation of unnecessary cellular molecules and organelles by delivering them to lysosomes, in which they are broken down by hydrolytic enzymes. Autophagy is also important as a defense system against pathogens as it can be involved in the degradation of proteins of invasive microbes. Three basic types of autophagy are distinguished: macroautophagy, chaperone-mediated autophagy (CMA), and microautophagy (reviewed in Mizushima and Komatsu (2011)). Macroautophagy is mediated by the formation of autophagosome, a double-membrane vacuole, containing materials to be degraded (cargo). Autophagosome combines with lysosome, resulting in a structure called autolysosome, in which the final degradation of the cargo occurs. Microautophagy is conceptually the simplest mechanism of autophagy as it involves engulfing of the cargo by the lysosome, initiated by the invagination of its membrane. Substrates for CMA are proteins with the KFERQ amino acid motif, which are unfolded by the HSC70 protein, a chaperone, assisted by several other proteins. Macroautophagy, which is considered as the major autophagic pathway and has been the most extensively studied among all types of autophagy, will be further referred to as autophagy. It involves a combined action of autophagosome and lysosome, in which the degrading action of lysosomal enzymes is supported by several autophagy-related proteins (Atgs) (reviewed in Blasiak et al. (2012)). The critical event in the mTOR (mammalian target of rapamycin)-dependent formation of autophagosome is the inactivation of mTOR, resulting in an immediate dephosphorylation of Atg13, activation of serine/threonine kinases (ULKs), and recruitment of the FIP-200 protein (Fig. 2a). Autophagy can be also initiated in various mTOR-independent mechanisms (reviewed in Sarkar (2013)). Detailed mechanisms of signaling in autophagy have been described in many excellent reviews (Boya et al. 2013; Gallagher et al. 2016; Yang and Klionsky 2010). Autophagosomes are formed in the vicinity of endoplasmic reticulum (ER), but it is not known, whether ER membrane directly participates in the development of autophagosome (reviewed in Mizushima et al. (2011)).

The involvement of autophagy in removing faulty cellular components makes it as an internal quality control mechanism in the cell. This has led to the concepts of general autophagy called non-selective autophagy and selective autophagy—the latter is directed to selective degradation of specific cellular components, first of all proteins or invading pathogens (reviewed in Mizushima and Komatsu (2011)). It is anticipated that even 1.5% of all cellular proteins in rat liver can be degraded by autophagy per hour in normal nutrition (Ezaki et al. 2011) (reviewed in Ueno et al. (2012)). Selective autophagy plays an important role in tissue differentiation, contributing to different transfer of genetic information in different cells. One of the most significant substrates for selective autophagy is the p62 protein (p62/SQSTM1), interacting with microtubule-associated protein light chain 3 (LC3), facilitating degradation of ubiquitinated substrates with its own subsequent degradation in autophagosome (reviewed in Johansen and Lamark (2011)). Therefore, p62 can be considered a marker of autophagic flux (Johansson et al. 2015; Viiri et al. 2013). The accumulation of p62 is associated with disturbed autophagy and observed in serious diseases including brain and liver cancers, Parkinson disease, Alzheimer disease, and steatohepatitis (Zatloukal et al. 2002). p62 decides the fate of the cell since it can be involved in pro-survival pathway by activation of the TRAF6 (TNF receptor-associated factor 6)-NF-κB pathway (Linares et al. 2013). On the other hand, p62 can activate caspase-8 through its polyubiquitination and association with death-inducing signaling complex (DISC), leading to cell death (Hughes et al. 2009; Singh et al. 2014). Various interactions of p62 include the stabilization of Nrf2 and activation of the transcription of Nrf2-related genes, including those which are crucial for cellular antioxidant defense (reviewed in Bryan et al. (2013)) (Johansson et al. 2015; Wang et al. 2014). Nrf2 was shown to transcriptionally regulate autophagy genes in a mouse model of Alzheimer disease (Pajares et al. 2016). As mentioned, to protect against oxidative stress, Nrf2 can cooperate with Keap1, but the activation of Nrf2 can promote autophagic degradation of Keap1 (Park et al. 2015). It seems that the role Nrf2 plays in autophagy, especially through the interaction with p62, strongly depends on the cellular context as there are many reports suggesting different actions of this protein in different cellular states (reviewed in Taniguchi et al. (2016)).

Autophagy plays a number of pro-survival functions in the cell (Fig. 2b). The production of amino acids is dependent on the availability of their components, which are normally supplied with nutrition, but in starvation, they can be delivered by increased autophagy, which results in increased amino acid production from degraded polypeptides (Vabulas and Hartl 2005). Amino acids can be included in the tricarboxylic acid (TCA) cycle to produce ATP, and autophagy was shown to play a role in this process by regulating the flow of metabolites in the TCA cycle (Guo et al. 2011). The recycling properties of autophagy—degrading of unneeded material to produce components for new blocks—make it a suitable tool for cell renovation during differentiation and development (reviewed in Mizushima and Komatsu (2011)). This is especially important just after fertilization, when the maternal material should be replaced by that encoded by the zygotic genome (Tsukamoto et al. 2008a, b). In that stage of development, autophagy plays an important role in establishing a maternal line of inheritance of mitochondrial DNA (mtDNA), since it is crucial for selective degradation of paternal mitochondria (reviewed in Sato and Sato (2013a, b)) (Zhou et al. 2011a, b). In general, degradation of mitochondria in the autophagic process is called mitophagy, and elimination of paternal mitochondria in early embryos by allogeneic organelle autophagy is called allophagy (reviewed in Sato and Sato (2013b)).

Results of several studies suggest that autophagy plays a role in the pathogenesis of various eye diseases, including those involving retinal degeneration (reviewed in Li et al. (2015)).

## Autophagy—an important player in the pathogenesis of age-related macular degeneration

**Fig. 3:**
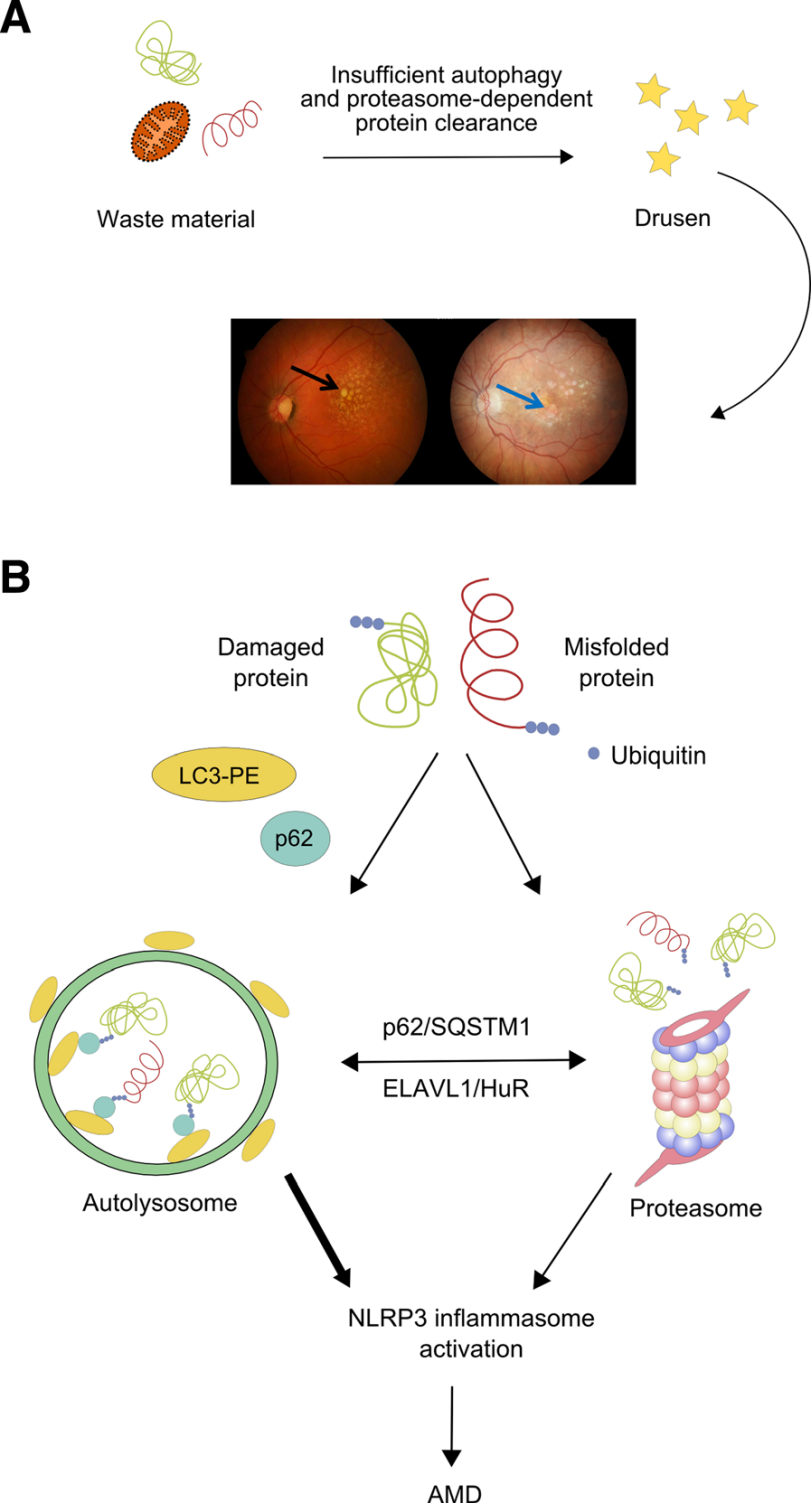
Extracellular drusen deposition can be detected in early dry age-related macular degeneration (AMD) (*black arrow*) if there is not sufficient protein clearance dependent on autophagy. Advanced dry AMD can lead to geographic atrophy with extensive cell death (*blue arrow*) (**a**). Protein waste material can be cleared by both autophagy and proteasomal degradation. These two pathways can cooperate and the interaction between p62/SQSTM1 and ELAVL1/HuR factors is critical for this cooperation. However, p62/SQSTM1 is preferentially accumulated over ELAVL1/Hur in AMD-inducing activation of the NLRP3 inflammasome, suggesting a greater contribution of impaired autophagy than proteasomal degradation in this disease (**b**)

As autophagy plays an important role in the homeostasis of RPE cells, its impairment can lead to accumulation of damaged organelles and various non-functional or toxic proteins, including lipofuscin, and promote the formation of drusen, typical for AMD. Drusen contains lipids, carbohydrates, proteins, and cellular debris, which are processed by autophagy. Therefore, impaired autophagy can contribute to drusen formation and in this way can be associated with AMD (Fig. 3a). However, no causative relationship follows from this association since there is no solid evidence that drusen can cause AMD (reviewed in Khan et al. (2016)). In general, a decrease of autophagic activity with age is observed, which can contribute to age-related diseases, including AMD (reviewed in Cuervo (2008) and Kaarniranta et al. (2013)) (Linares et al. 2013). However, it is not exactly known which autophagic pathway contributes mostly to this effect. Moreover, the role of different autophagic mechanisms in the retinal physiology and pathology is not completely clear (Rodriguez-Muela et al. 2013). However, the relationship between the presence of drusen in AMD and autophagy impairment can be seen in a wider context, as age-related dysfunction of autophagy can lead to the formation and accumulation of drusen, containing cellular debris, lipids, carbohydrates, and proteins that are believed to be delivered from the RPE cells. Therefore, the association between different autophagic pathways and drusen formation could be informative for the contribution of these processes to AMD biogenesis and progression.

Not only autophagy but also proteosomal-mediated proteolysis can play a role in AMD. We showed that the expression of p62/SQSTM1 was post-transcriptionally regulated by the ELAVL1/HuR factor, when proteosomal activity was inhibited in ARPE-19 cells (Viiri et al. 2013). Therefore, p62/SQSTM1 can be considered a central protein for the involvement of disturbed autophagy and proteolysis in drusen formation and the role of this effect in AMD pathogenesis. Samples from AMD patients showed a stronger accumulation of p62/SQSTM1 rather than ELAVL1/HuR, suggesting a greater contribution of autophagy in clearing protein aggregates than proteasome-mediated proteolysis, and in this way, autophagy can be seen as the main mechanism involved in the protein aggregation in RPE cells (Fig. 3b). Since increased protein aggregation can contribute to inflammasome activation and tissue injury, it may be speculated that decreased autophagy and increased inflammation participate in drusen biogenesis (Liu et al. 2016; Wang et al. 2014) (reviewed in Wang et al. (2016a, b)).

Mitter and coworkers observed over 100% increase in the number of autophagosomes in RPE of two mouse models of AMD and human AMD donors (Mitter et al. 2014). A significant increase in the expression of the LC3, Atg7, and Atg9 proteins was observed in SOD2-knocked mice in their early stage of development. Similar increase was noted in the tissue sections from AMD donors. However, both experimental animals and AMD donors showed a decreased autophagosome fractional volume in both early and late stages of AMD as compared to controls. The results obtained in that work are important for several reasons. Firstly, increasing autophagy with age in mice RPE is somehow surprising in the context of general decrease of autophagy with age. However, at this point, two features should be distinguished: autophagy potential and autophagy activity. One can assume that overall autophagy capacity decreases with age, but this does not exclude that there is an increased autophagy flux in the initial phase of oxidative stress, when a large amount of damaged proteins and other molecules is produced. On the other hand, this can lead to overloading of the autophagosomal system, resulting in a decrease in autophagy activity. These effects can be associated with initial phase of AMD. The authors showed a dynamic character of autophagy in RPE cells in oxidative stress, which was displayed in increased and decreased autophagy evoked by acute and chronic stress, respectively. The initial increase in autophagy was attributed to increased number of autophagosomes, which can result from a reduced effectiveness of lysosomes to degrade autophagosomal content. However, the authors did not directly check this possibility, as it is technically challenging, but this feature can be associated with AMD (reviewed in Kaarniranta et al. (2013) and Mitter et al. (2012)).

Five autoreactivity targets were found in the age-related maculopathy ancillary (ARMA) study to identify and characterize human macula autoantigens in sera of AMD subjects: two belonging to heat shock protein 70 (HSP70) family HSPA8/9, another heat shock protein HSPB4, also known as alpha-crystallin A chain (CRYAA), Annexin A5, and Protein S100-A9 (Iannaccone et al. 2015). Although these proteins can be involved in various effects and mechanisms important for AMD development, including immunomodulation and antioxidative and anti-apoptotic protection, they all can participate in autophagy activation, confirming an important role of this process in AMD pathogenesis.

The critical role of autophagy in the homeostasis of aging RPE was shown in mice with knockout in the gene coding for the RB1CC1/FIP200 protein, which is essential for autophagy induction (Yao et al. 2015). These mice developed age-related degeneration of RPE, which was featured by atrophic patches, subretinal migration of microglia, deposition of oxidatively damaged proteins, and choroidal neovascularization. Degenerated RPE was linked with the loss of neighboring photoreceptors. Although it is known that A2E, a component of lipofuscin exerting a toxic effect on RPE cells, is deposited in these cells with age, its exact mechanism contributing to AMD pathogenesis is not completely known. It was shown that A2E induced autophagy and decreased the viability of RPE cells, indicating another mechanism of the involvement of autophagy to AMD pathogenesis (Guha et al. 2013; Zhang et al. 2015). In general, the interrelationships between autophagy-lysosomal and the ubiquitin-proteosome pathways can be important for the pathogenesis of AMD, in which accumulation of non-functional proteins is observed (Zhan et al. 2016). It was observed that impaired autophagy resulted in a decreased proteosomal-mediated proteolysis, suggesting that autophagy can be a principal system for clearance of misfolded and otherwise damaged proteins in human RPE cells.

Oxidative stress plays a role in many aspects of AMD pathogenesis, and cellular antioxidant response, which can be seen as an AMD protective mechanism, is linked to autophagy through the p62/Keap1/Nrf2 pathway (Johansson et al. 2015; Wang et al. 2014). However, interconnections between autophagy, oxidative stress, and AMD can be wider as autophagy can be associated with other elements of antioxidant defense, including DNA repair (reviewed in Czarny et al. (2015) and Filomeni et al. (2015)). As DNA damage response (DDR) can be important for AMD, its association with autophagy can play a role in AMD pathogenesis. As we showed, variability of DNA repair genes, including the base excision repair pathway *hOGG1* and *MUTYH* genes, was associated with AMD (reviewed in Blasiak et al. (2012)) (Synowiec et al. 2012). However, the exact role of autophagy in DDR or rather an association between these two phenomena was not determined. Likely, the most direct link between them is expressed by mitophagy, when mitochondria with highly damaged DNA are degraded (Kurihara et al. 2012). This is a specific feature of DDR in mitochondria, as in the nucleus, seriously damaged DNA can induce apoptosis or, in certain circumstances, be tolerated resulting in mutations (reviewed in Sharma et al. (2013)). Although it is known that reactive oxygen species (ROS) can induce autophagy in starvation conditions, it is not known which exactly species are responsible for this effect (reviewed in Filomeni et al. (2010)). Both superoxide radical (O_2_^•–^) and hydrogen peroxide (H_2_O_2_) were considered species involved in autophagy triggering in starvation conditions (Chen et al. 2009; Scherz-Shouval et al. 2007a; Scherz-Shouval et al. 2007b; Zhang et al. 2013). In general, ROS can be considered inducers of autophagy (reviewed in Azad et al. (2009; Levonen et al. 2014). Moreover, some data suggest that mitochondria are the main source of ROS needed for the induction of autophagy (Scherz-Shouval et al. 2007a; Scherz-Shouval et al. 2007b) (reviewed in Scherz-Shouval and Elazar (2007)). Therefore, it seems that there is a logical chain of events: starvation increases energetic demands, which in turn increase ATP production in mitochondria and elevate ROS level. However, the action of ROS, especially in oxidative stress, can induce also other mechanisms, which can interfere with autophagy.

A diet rich in polyunsaturated fatty acid (PUFA) is considered an AMD risk factor, but the question whether it is an independent factor is not completely answered (reviewed in Lambert et al. (2016)). However, one of the mechanisms underlying this association can result from the peroxidation of PUFA as it results in the formation of lipid peroxidation products, which can be detrimental for the cell (reviewed in Ayala et al. (2014)). This effect can be associated with wet AMD as it was observed that malondialdehyde (MDA), a major lipid peroxidation product, induced VEGF secretion, which was associated with choroidal neovascularization, typical for wet AMD (Ye et al. 2016). Moreover, MDA also induced autophagy impairment in AMD patients and ARPE-19 cells, observed as changes in the expression of Beclin 1, LC3B, and p62 proteins. Therefore, MDA accumulation can be an important step in AMD pathogenesis related to oxidative stress and autophagy.

## Autophagy, apoptosis, pyroptosis, and necroptosis in AMD

Autophagy improves cell survival through the recycling of metabolic precursors and removing damaged proteins and organelles from the cell. Additionally, autophagy modulates inflammation responses and cell death pathways and thereby influences disease pathogenesis, including AMD (reviewed in Kaarniranta et al. (2013) and Nikoletopoulou et al. (2013)). Autophagy inhibition resulted in inflammasome activation and increased angiogenesis in RPE cells exposed to rotenone, an inhibitor of the electron transport chain in mitochondria (Liu et al. 2016). Inhibited autophagy induced caspase-3-mediated cell death, suggesting that apoptosis can be over positive control of autophagy in RPE cells.

Hydrogen peroxide at high concentrations induced necrotic cell death, mediated by the activation of poly(ADP-ribose) polymerase 1 (PARP1) in human RPE cells in culture, and nicotinamide adenine dinucleotide (NAD^+^) protected the cells against this effect (Zhu et al. 2016). Using autophagy inhibitors, it was shown that autophagy was responsible for that protective effect. Therefore, two important compounds can play a role in the regulation of cellular death in AMD: NAD^+^ and PARP1, and their action can be related to autophagy. Ultrastructural pathology studies suggest that the predominant mechanisms of cellular death in AMD were pyroptosis and necroptosis, while apoptosis could have a minor contribution (reviewed in Ardeljan et al. (2014)).

Mitochondria seem to be an important element of regulation of cellular death in AMD for several reasons. Firstly, they are important for the intrinsic apoptosis pathway (Criollo et al. 2007) (reviewed in Kroemer et al. (2007)). Secondly, they are linked to inflammatory response and pyroptosis (Zhou et al. 2011a, b). Thirdly, the process of cell degeneration, crucial for acquiring AMD phenotype, can be associated with dysfunctional mitochondria (reviewed in Carelli et al. (2002)). Autophagy proteins can inhibit inflammasome activation by reducing the release of mitochondrial DNA (Nakahira et al. 2011).

As AMD is associated with inflammation, pyroptosis could be initiated at its specific level. This was confirmed by the presence of caspase-1 in the NLRP3 inflammasome (Wang et al. 2016a, b). Results of some studies suggest that pyroptosis can be functionally linked with autophagy. It was shown that inhibition of autophagy stimulated pneumococcus-induced pyroptosis and protected microglia cells against pyroptosis (Kim et al. 2015). Extensive studies led by Hanus et al. (2015b) suggest that necroptosis can dominate in RPE cell death associated with dry AMD.

**Fig. 4:**
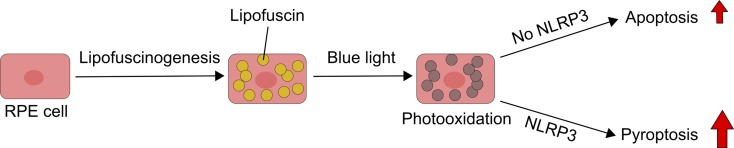
Pyroptosis can dominate over apoptosis in retinal pigment epithelium (RPE) cells containing lipofuscin and irradiated with blue light, when these processes induce activation of the NLRP3 inflammasome

An interplay between apoptosis and pyroptosis was observed in research, in which ARPE-19 cells and primary human RPE cells were loaded with lipofuscin and irradiated with blue light (Brandstetter et al. 2016) (Fig. 4). The irradiated lipofuscin-mediated oxidative stress resulted in damage to the lysosomal membrane and leakage of lysosomal enzymes into the cytosol and eventually cell death by apoptosis. However, this mode of cellular death was changed into pyroptosis after the NLRP3 inflammasome priming by IL-1α or C5a, a complement activation product, or conditioned media for pyroptotic cells. Activation of the NLRP3 inflammasome is an important mechanism regulating cell death in RPE, and it was shown to occur in AMD-affected RPE cells (Gelfand et al. 2015; Tseng et al. 2013). Therefore, results of these studies suggest that inflammasome activation alters the preferable cell death mode induced by photooxidation from apoptosis to pyroptosis.

**Fig. 5:**
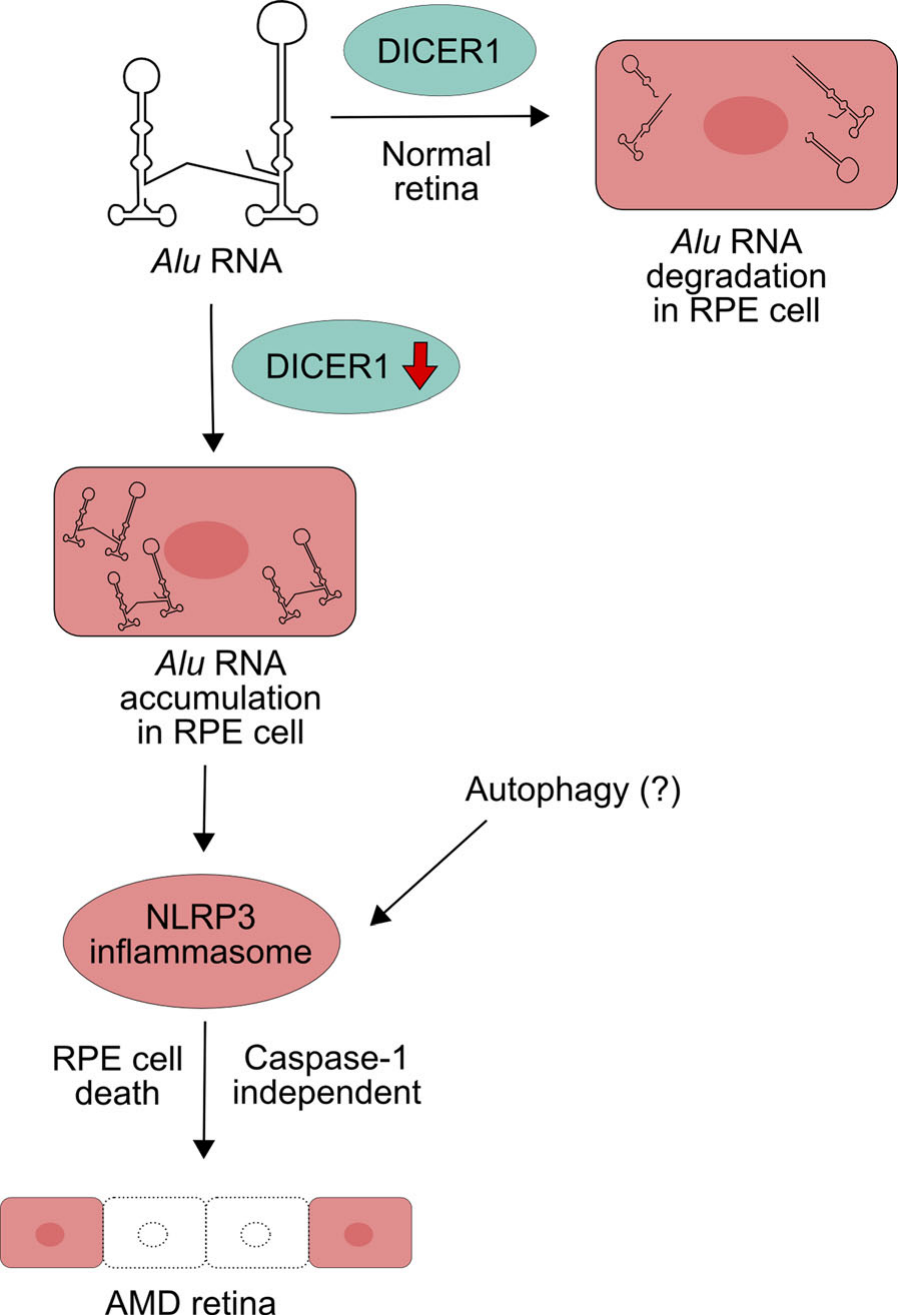
Normally, *Alu* RNAs, produced in the transcription of the *Alu* transposons, are degraded by the DICER1 endonuclease, but if its activity decreases, there is an accumulation of the *Alu* RNAs, which, in turn, induces the NLRP3 inflammasome resulting in a caspase-1-independent RPE cell death. The last event can be modulated by autophagy

The *Alu* transposons are common in the human genome, and the amount of their transcripts is regulated by the DICER1 RNase, which activity was reported to decrease and result in accumulation of the transcripts in the eye of patients with AMD (Kaneko et al. 2011) (Fig. 5). These RNAs are toxic for RPE and can induce the death of RPE cells (Tarallo et al. 2012). Tarallo et al. (2012) developed a concept that *Alu* RNAs were recognized by an innate immune system. They showed that *Alu* RNA accumulation, likewise DICER1 deficiency, activated the NLRP3 inflammasome, and this activation was independent of toll-like receptors (TLRs), a major class of innate immune receptors, which can be involved in the recognition of both single- and double-stranded RNAs, present in *Alu* RNA transposons (Sinnett et al. 1991). In general, this mechanism critically depends on the NF-kB and P2X7 signaling intermediates (Kerur et al. 2013). Therefore, *Alu* RNA accumulation in non-immune RPE cells can lead to the NLRP3 inflammasome activation and RPE cell death, but the kind of the death is unknown. However, pyroptosis was excluded, despite activation of caspase-1 by *Alu* RNAs. As DICER1 displays anti-apoptotic properties, it can be speculated that its deficiency can stimulate apoptotic mechanisms of cell death (Nekova et al. 2014). This was confirmed by observation that caspase-8 is a critical mediator of the degeneration of RPE cells associated with DICER1 deficiency and *Alu* RNA accumulation (Kim et al. 2014). The enzyme was activated through the Fas ligand-dependent pathway, which is typical for the extrinsic mode of apoptosis. It was reported that retrotransposons localized in RNA granules were selectively degraded with the involvement of the NDP52 and p62 proteins, which are autophagy receptors (Guo et al. 2014). Moreover, mice with inactivated Atg6/Beclin1, essential for autophagy, accumulated retrotransposon RNA and insertions in their genomes. Therefore, one can speculate that impaired autophagy can play a role in AMD pathogenesis also by supporting *Alu* RNA accumulation.

The involvement of DICER1 in the degeneration of RPE cells can be independent of its role in microRNA (miRNA) biogenesis. However, it was reported that DICER1 and the EIF2CA/AGO complex, which is essential for miRNA homeostasis, could be degraded by autophagy, if not loaded with miRNA (reviewed in Gibbings et al. (2013)). The degradation resulted from the recognition by the selective autophagy receptor CALCOCO2/NDP52 (calcium-binding and coiled-coil domain 2/nuclear dot protein, 52 kDa). Therefore, autophagy can be important in the regulation of the pool of miRNA-free DICER1, which could target *Alu* RNA, and this mechanism can contribute to AMD pathogenesis. Several interconnections between autophagy and miRNA biogenesis were reported in cancer and other diseases and aging, so it would be useful to relate these results to AMD (Bao et al. 2016; Tan et al. 2016b; Zhang et al. 2016).

## Conclusions and perspectives

AMD is a major eye disease and emerging health problem in developed countries with no remedy in general. Studies on the mechanisms of AMD pathogenesis are crucial for effective treatment of this disease. As AMD is a degenerative disease, cell death underlies its phenotypic expression. However, the precise mechanism regulating the death of RPE cells and photoreceptors, crucial for AMD progression, is not known. It should be taken into account that such mechanism could not be represented by a simple chain of events, as AMD is a highly non-homogenous disease with various phenotypes and severity. The most basic question is whether AMD-affected cells undergo programmed death or necrosis? Many reports indicate necrosis as the main mode of RPE cell death in AMD, but some of them describe rather its programmed version, necroptosis, than fully uncontrolled cell death (reviewed in Galluzzi et al. (2011) and Vandenabeele et al. (2010)) (Holler et al. 2000; Murakami et al. 2014). Therefore, programmed cellular death should be rather linked with AMD pathogenesis.

**Fig. 6:**
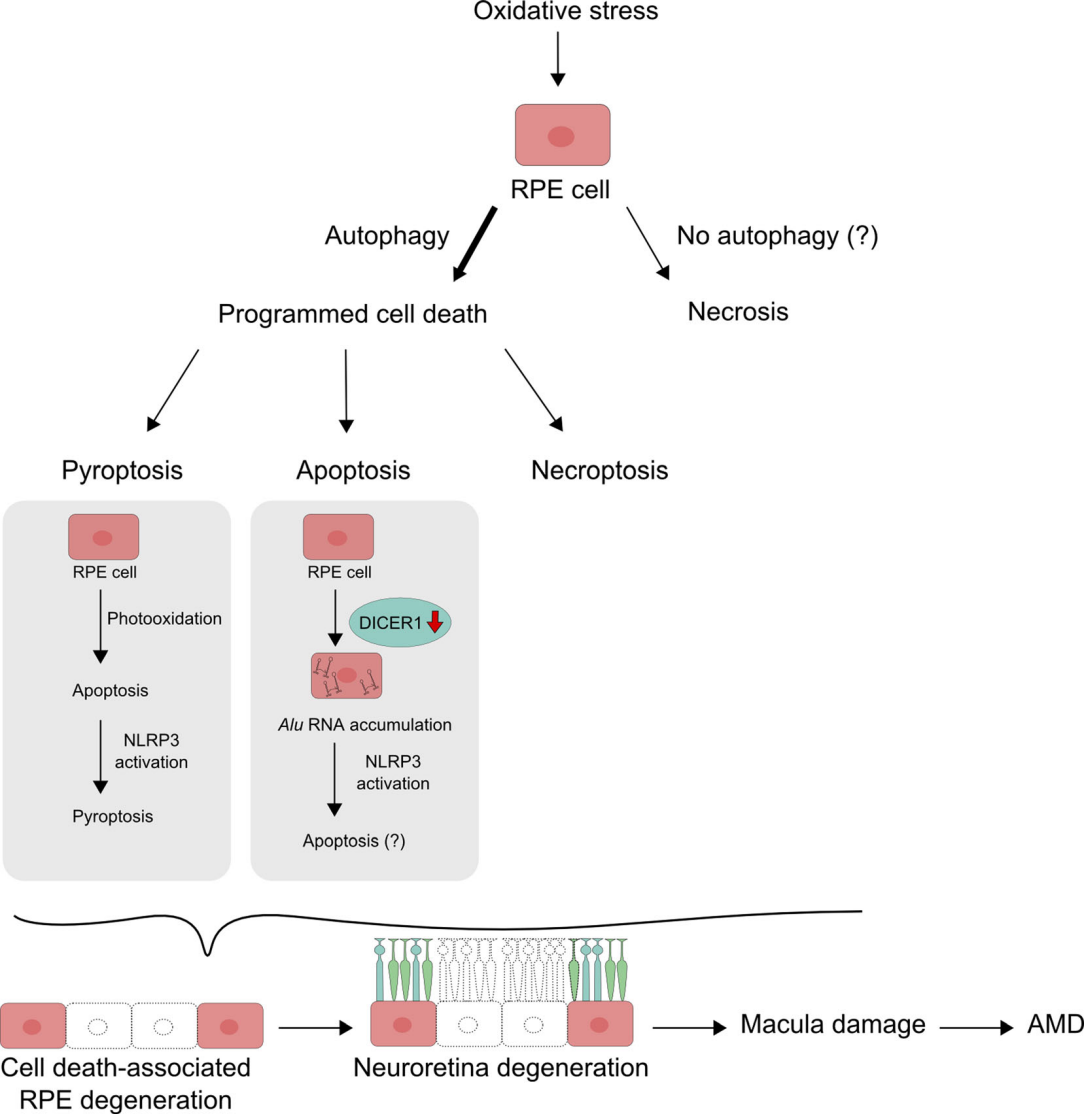
Retinal pigment epithelium (RPE) cells undergoing oxidative stress, which is causative for age-related macular degeneration (AMD), can die either by necrosis with no involvement of autophagy or by a programmed cell death regulated by autophagy. Various mechanisms decide about the mode of cell death. When oxidative stress is generated by blue light, it can induce apoptosis, but if it is associated with the activation of the NLRP3 inflammasome, pyroptosis can dominate. When oxidative stress results in DICER1 activity reduction and/or *Alu* RNA accumulation, it can induce a caspase-1-independent cell death, likely apoptosis

Apoptosis is a cell death, which is most often attributed to AMD. However, similar to necrosis and necroptosis, some reports indicating apoptotic cell death in AMD could in fact report pyroptosis instead. As inflammation can be strongly associated with AMD, pyroptosis could be a preferential consequence of that association. Therefore, necroptosis, apoptosis, and pyroptosis can be considered the main death modes occurring in RPE cells in AMD (Fig. 6). Executory pathways of these phenomena are generally independent of each other, so the choice of one of them can determine the fate of the cell. Therefore, the mechanism of the choice is important for therapeutic strategies in AMD as it can be targeted to inhibit degeneration of the retina through the inhibition or delay of death of RPE cells.

Exploring the role autophagy plays in AMD pathogenesis, it would be justified to look for the association between known AMD susceptibility genes and autophagy. Genome-wide association study (GWAS) as well as earlier studies on AMD genetics revealed many genes, which variability can be associated with AMD occurrence and progression. GWAS and Exome-Chip attitudes allowed for the identification of more than 50 common and rare variants at more than 30 loci (Fritsche et al. 2016). They can be classified in several ways. One of them categorizes AMD susceptibility genes into five main categories: inflammation and immune response, cell stress response, lipid metabolism and transport, extracellular matrix and cell adhesion, and angiogenesis (reviewed in Tan et al. (2016a, b)). It should be noted that the causative role of these genes in AMD pathology is not fully known. Whether any of these genes can be closely related to autophagy is an open question. We showed that ADAMTS9 locus can be considered a candidate for such an association (Helisalmi et al. 2014). However, although there is no solid evidence for link of autophagy with AMD susceptibility, some autophagy-related genes can play a role in AMD pathogenesis as they are important for some general phenomena occurring in the AMD course, and in this review, we tried to show that autophagy can be involved in AMD-associated RPE cell death.

**Fig. 7:**
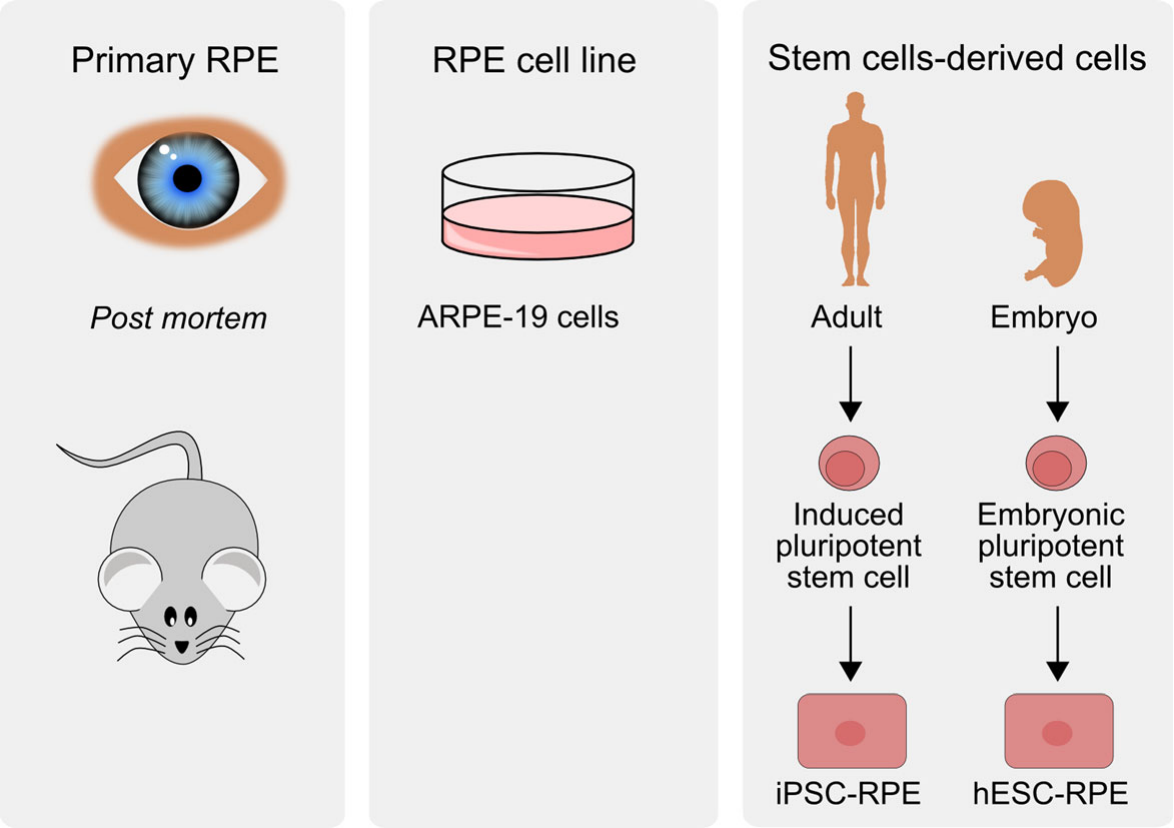
Several experimental in vitro models to study molecular aspects of the pathogenesis of age-related macular degeneration have been established. ARPE-19 cells are an example of cell cultures derived from retinal pigment epithelium (RPE). Primary RPE cells can be obtained post-mortem from humans or laboratory animals with transgenic modification. Embryonic pluripotent stem cells or induced pluripotent stem cells obtained from individuals with AMD susceptibility differentiated into RPE cells can contribute to a consistent model of AMD

At present, there is no appropriate model of AMD. Human live experiments are excluded, and while there are many animal models of AMD, no one reflects all features of human AMD and they have probably more limitations than strengths (Lyzogubov et al. 2016). Although stem cell cultures are employed in research on molecular pathogenesis of AMD, the majority of in vitro studies on retinal cell death in AMD are performed on RPE-derived cell cultures, mostly the ARPE-19 cell line (Hsiung et al. 2015) (Fig. 7). There are many experimental limitations to study cell death in ARPE-19 cells, including heterogeneity of different clones in different laboratories. Moreover, some studies were performed on cell lines with genetic manipulations, additionally contributing to that heterogeneity. Last, but not the least, AMD is a systemic disease and not just an RPE disorder. Nevertheless, detailed studies on mechanism of cellular death in AMD may be performed only on cell in culture. However, there are at least two other major problems associated with the use of cell lines. Firstly, AMD is an age-related disease and RPE cells in the macula are post-mitotic, so their aging in situ may differ from this process occurring in a culture. Moreover, ARPE-19 populations can contain a substantial, if not major, fraction of cells which are able to double their population to over 270 times, so they can be considered immortal, and mechanisms of cell death in mortal and immortal cells can be different (Kozlowski 2015). Furthermore, senescence may not be the only reason for the difference between RPE cells in vivo and in vitro and internal heterogeneity within ARPE-19 population (reviewed in Kozlowski (2012)). Secondly, the exact mechanism of triggering cellular death in AMD, leading to macular degeneration, is not completely known; thus, a study of death in cell culture is somehow arbitrary as it can depend on the factor to induce that effect.

Therapeutic strategy targeting the mechanism regulating RPE cell death would be useful as it could be a universal strategy for AMD. At present, the only effective treatment for AMD is based on the use of anti-VEGF in the wet form of the disease. There is no remedy for GA in the dry AMD. However, as we tried to stress in our review, the mechanism of determination of RPE cell death is far from completeness. We tried to show that autophagy was involved in most cases of AMD-related death of RPE cells. This involvement is underlined by the role inflammation plays in AMD pathogenesis and regulation of the NLRP3 inflammasome, playing a central role in both dry and wet forms of the disease, by autophagy. Therefore, research on the inhibition of NLRP3 activation by autophagy proteins should search for detailed mechanisms of this interaction, projecting some chemical or/and genetic modulators of this interaction.

## Abbreviations

AIF: Apoptosis-inducing factor
AMD: Age-related macular degeneration
ARMA: Age-related maculopathy ancillary
Atg: Autophagy-related protein
CALCOCO2/NDP52: Calcium-binding and coiled-coil domain 2/nuclear dot protein, 52 kDa
CMA: Chaperone-mediated autophagy
CRYAA: Alpha-crystallin A chain
DDR: DNA damage response
DISC: Death-inducing signaling complex
ER: Endoplasmic reticulum
GA: Geographic atrophy
GWAS: Genome-wide association study
Keap1: Kelch-like ECH-associated protein 1
LC3: Microtubule-associated protein light chain 3
MDA: Malondialdehyde
Nrf2: Nuclear factor (erythroid-derived 2)-like 2
p62/SQSTM1: Sequestome-1
PARP1: Poly(ADP-ribose) polymerase 1
PUFA: Polyunsaturated fatty acid
RPE: Retinal pigment epithelium
SOD: Superoxide dismutase
TCA: Tricarboxylic acid
TRAF6: TNF receptor-associated factor 6
ULK: Serine/threonine kinase.

## References

[CR1] Adler R, Curcio C, Hicks D, Price D, Wong F (1999). Cell death in age-related macular degeneration. Mol Vis.

[CR2] Ardeljan CP, Ardeljan D, Abu-Asab M, Chan CC (2014). Inflammation and cell death in age-related macular degeneration: an immunopathological and ultrastructural model. J Clin Med.

[CR3] Ayala A, Munoz MF, Arguelles S (2014). Lipid peroxidation: production, metabolism, and signaling mechanisms of malondialdehyde and 4-hydroxy-2-nonenal. Oxidative Med Cell Longev.

[CR4] Azad MB, Chen Y, Gibson SB (2009). Regulation of autophagy by reactive oxygen species (ros): implications for cancer progression and treatment. Antioxid Redox Signal.

[CR5] Bao L, Lv L, Feng J, Chen Y, Wang X, Han S, Zhao H. Mir-487b-5p regulates temozolomide resistance of lung cancer cells through lamp2-medicated autophagy. DNA Cell Biol. 2016;35:385–92. 10.1089/dna.2016.3259 27097129

[CR6] Blasiak J, Synowiec E, Salminen A, Kaarniranta K (2012). Genetic variability in DNA repair proteins in age-related macular degeneration. Int J Mol Sci.

[CR7] Boya P, Reggiori F, Codogno P (2013). Emerging regulation and functions of autophagy. Nat Cell Biol.

[CR8] Brandstetter C, Patt J, Holz FG, Krohne TU (2016). Inflammasome priming increases retinal pigment epithelial cell susceptibility to lipofuscin phototoxicity by changing the cell death mechanism from apoptosis to pyroptosis. J Photochem Photobiol B.

[CR9] Bryan HK, Olayanju A, Goldring CE, Park BK (2013). The nrf2 cell defence pathway: Keap1-dependent and -independent mechanisms of regulation. Biochem Pharmacol.

[CR10] Carelli V, Ross-Cisneros FN, Sadun AA (2002). Optic nerve degeneration and mitochondrial dysfunction: genetic and acquired optic neuropathies. Neurochem Int.

[CR11] Chen Y, Azad MB, Gibson SB (2009). Superoxide is the major reactive oxygen species regulating autophagy. Cell Death Differ.

[CR12] Criollo A, Galluzzi L, Maiuri MC, Tasdemir E, Lavandero S, Kroemer G (2007). Mitochondrial control of cell death induced by hyperosmotic stress. Apoptosis.

[CR13] Cuervo AM (2008). Autophagy and aging: keeping that old broom working. Trends Genet.

[CR14] Czarny P, Pawlowska E, Bialkowska-Warzecha J, Kaarniranta K, Blasiak J (2015). Autophagy in DNA damage response. Int J Mol Sci.

[CR15] Dunaief JL, Dentchev T, Ying GS, Milam AH (2002). The role of apoptosis in age-related macular degeneration. Arch Ophthalmol.

[CR16] Ezaki J, Matsumoto N, Takeda-Ezaki M, Komatsu M, Takahashi K, Hiraoka Y, Taka H, Fujimura T, Takehana K, Yoshida M, Iwata J, Tanida I, Furuya N, Zheng DM, Tada N, Tanaka K, Kominami E, Ueno T (2011). Liver autophagy contributes to the maintenance of blood glucose and amino acid levels. Autophagy.

[CR17] Ferrington DA, Sinha D, Kaarniranta K (2016). Defects in retinal pigment epithelial cell proteolysis and the pathology associated with age-related macular degeneration. Prog Retin Eye Res.

[CR18] Filomeni G, Desideri E, Cardaci S, Rotilio G, Ciriolo MR (2010). Under the ros...thiol network is the principal suspect for autophagy commitment. Autophagy.

[CR19] Filomeni G, De Zio D, Cecconi F (2015). Oxidative stress and autophagy: the clash between damage and metabolic needs. Cell Death Differ.

[CR20] Fritsche LG, Igl W, Bailey JN, Grassmann F, Sengupta S, Bragg-Gresham JL, Burdon KP, Hebbring SJ, Wen C, Gorski M, Kim IK, Cho D, Zack D, Souied E, Scholl HP, Bala E, Lee KE, Hunter DJ, Sardell RJ, Mitchell P, Merriam JE, Cipriani V, Hoffman JD, Schick T, Lechanteur YT, Guymer RH, Johnson MP, Jiang Y, Stanton CM, Buitendijk GH, Zhan X, Kwong AM, Boleda A, Brooks M, Gieser L, Ratnapriya R, Branham KE, Foerster JR, Heckenlively JR, Othman MI, Vote BJ, Liang HH, Souzeau E, McAllister IL, Isaacs T, Hall J, Lake S, Mackey DA, Constable IJ, Craig JE, Kitchner TE, Yang Z, Su Z, Luo H, Chen D, Ouyang H, Flagg K, Lin D, Mao G, Ferreyra H, Stark K, von Strachwitz CN, Wolf A, Brandl C, Rudolph G, Olden M, Morrison MA, Morgan DJ, Schu M, Ahn J, Silvestri G, Tsironi EE, Park KH, Farrer LA, Orlin A, Brucker A, Li M, Curcio CA, Mohand-Said S, Sahel JA, Audo I, Benchaboune M, Cree AJ, Rennie CA, Goverdhan SV, Grunin M, Hagbi-Levi S, Campochiaro P, Katsanis N, Holz FG, Blond F, Blanche H, Deleuze JF, Igo RP, Truitt B, Peachey NS, Meuer SM, Myers CE, Moore EL, Klein R, Hauser MA, Postel EA, Courtenay MD, Schwartz SG, Kovach JL, Scott WK, Liew G, Tan AG, Gopinath B, Merriam JC, Smith RT, Khan JC, Shahid H, Moore AT, McGrath JA, Laux R, Brantley MA, Agarwal A, Ersoy L, Caramoy A, Langmann T, Saksens NT, de Jong EK, Hoyng CB, Cain MS, Richardson AJ, Martin TM, Blangero J, Weeks DE, Dhillon B, van Duijn CM, Doheny KF, Romm J, Klaver CC, Hayward C, Gorin MB, Klein ML, Baird PN, den Hollander AI, Fauser S, Yates JR, Allikmets R, Wang JJ, Schaumberg DA, Klein BE, Hagstrom SA, Chowers I, Lotery AJ, Leveillard T, Zhang K, Brilliant MH, Hewitt AW, Swaroop A, Chew EY, Pericak-Vance MA, DeAngelis M, Stambolian D, Haines JL, Iyengar SK, Weber BH, Abecasis GR, Heid IM (2016). A large genome-wide association study of age-related macular degeneration highlights contributions of rare and common variants. Nat Genet.

[CR21] Gallagher LE, Williamson LE, Chan EY. Advances in autophagy regulatory mechanisms. Cell. 2016;5(2)24. doi:10.3390/cells5020024 10.3390/cells5020024 PMC4931673 27187479

[CR22] Galluzzi L, Vanden Berghe T, Vanlangenakker N, Buettner S, Eisenberg T, Vandenabeele P, Madeo F, Kroemer G (2011). Programmed necrosis from molecules to health and disease. Int Rev Cell Mol Biol.

[CR23] Gelfand BD, Wright CB, Kim Y, Yasuma T, Yasuma R, Li S, Fowler BJ, Bastos-Carvalho A, Kerur N, Uittenbogaard A, Han YS, Lou D, Kleinman ME, McDonald WH, Nunez G, Georgel P, Dunaief JL, Ambati J (2015). Iron toxicity in the retina requires Alu RNA and the nlrp3 inflammasome. Cell Rep.

[CR24] Gibbings D, Mostowy S, Voinnet O (2013). Autophagy selectively regulates miRNA homeostasis. Autophagy.

[CR25] Guha S, Baltazar GC, Coffey EE, Tu LA, Lim JC, Beckel JM, Patel S, Eysteinsson T, Lu W, O'Brien-Jenkins A, Laties AM, Mitchell CH (2013). Lysosomal alkalinization, lipid oxidation, and reduced phagosome clearance triggered by activation of the p2x7 receptor. FASEB J.

[CR26] Guo H, Chitiprolu M, Gagnon D, Meng L, Perez-Iratxeta C, Lagace D, Gibbings D (2014). Autophagy supports genomic stability by degrading retrotransposon RNA. Nat Commun.

[CR27] Guo JY, Chen HY, Mathew R, Fan J, Strohecker AM, Karsli-Uzunbas G, Kamphorst JJ, Chen G, Lemons JM, Karantza V, Coller HA, Dipaola RS, Gelinas C, Rabinowitz JD, White E (2011). Activated Ras requires autophagy to maintain oxidative metabolism and tumorigenesis. Genes Dev.

[CR28] Hanus J, Zhang H, Wang Z, Liu Q, Zhou Q, Wang S (2013). Induction of necrotic cell death by oxidative stress in retinal pigment epithelial cells. Cell Death Dis.

[CR29] Hanus J, Anderson C, Wang S (2015). RPE necroptosis in response to oxidative stress and in AMD. Ageing Res Rev.

[CR30] Hanus J, Kolkin A, Chimienti J, Botsay S, Wang S (2015). 4-Acetoxyphenol prevents rpe oxidative stress-induced necrosis by functioning as an nrf2 stabilizer. Invest Ophthalmol Vis Sci.

[CR31] Helisalmi S, Immonen I, Losonczy G, Resch MD, Benedek S, Balogh I, Papp A, Berta A, Uusitupa M, Hiltunen M, Kaarniranta K (2014). Adamts9 locus associates with increased risk of wet AMD. Acta Ophthalmol.

[CR32] Holler N, Zaru R, Micheau O, Thome M, Attinger A, Valitutti S, Bodmer JL, Schneider P, Seed B, Tschopp J (2000). Fas triggers an alternative, caspase-8-independent cell death pathway using the kinase rip as effector molecule. Nat Immunol.

[CR33] Hsiung J, Zhu D, Hinton DR (2015). Polarized human embryonic stem cell-derived retinal pigment epithelial cell monolayers have higher resistance to oxidative stress-induced cell death than nonpolarized cultures. Stem Cells Transl Med.

[CR34] Hughes MA, Harper N, Butterworth M, Cain K, Cohen GM, MacFarlane M (2009). Reconstitution of the death-inducing signaling complex reveals a substrate switch that determines cd95-mediated death or survival. Mol Cell.

[CR35] Iannaccone A, Giorgianni F, New DD, Hollingsworth TJ, Umfress A, Alhatem AH, Neeli I, Lenchik NI, Jennings BJ, Calzada JI, Satterfield S, Mathews D, Diaz RI, Harris T, Johnson KC, Charles S, Kritchevsky SB, Gerling IC, Beranova-Giorgianni S, Radic MZ (2015). Circulating autoantibodies in age-related macular degeneration recognize human macular tissue antigens implicated in autophagy, immunomodulation, and protection from oxidative stress and apoptosis. PLoS One.

[CR36] Jing K, Lim K (2012). Why is autophagy important in human diseases?. Exp Mol Med.

[CR37] Johansen T, Lamark T (2011). Selective autophagy mediated by autophagic adapter proteins. Autophagy.

[CR38] Johansson I, Monsen VT, Pettersen K, Mildenberger J, Misund K, Kaarniranta K, Schonberg S, Bjorkoy G (2015). The marine n-3 PUFA DHA evokes cytoprotection against oxidative stress and protein misfolding by inducing autophagy and NFE2L2 in human retinal pigment epithelial cells. Autophagy.

[CR39] Kaarniranta K, Sinha D, Blasiak J, Kauppinen A, Vereb Z, Salminen A, Boulton ME, Petrovski G (2013). Autophagy and heterophagy dysregulation leads to retinal pigment epithelium dysfunction and development of age-related macular degeneration. Autophagy.

[CR40] Kaneko H, Dridi S, Tarallo V, Gelfand BD, Fowler BJ, Cho WG, Kleinman ME, Ponicsan SL, Hauswirth WW, Chiodo VA, Kariko K, Yoo JW, Lee DK, Hadziahmetovic M, Song Y, Misra S, Chaudhuri G, Buaas FW, Braun RE, Hinton DR, Zhang Q, Grossniklaus HE, Provis JM, Madigan MC, Milam AH, Justice NL, Albuquerque RJ, Blandford AD, Bogdanovich S, Hirano Y, Witta J, Fuchs E, Littman DR, Ambati BK, Rudin CM, Chong MM, Provost P, Kugel JF, Goodrich JA, Dunaief JL, Baffi JZ, Ambati J (2011). Dicer1 deficit induces Alu RNA toxicity in age-related macular degeneration. Nature.

[CR41] Kauppinen A, Paterno JJ, Blasiak J, Salminen A, Kaarniranta K (2016). Inflammation and its role in age-related macular degeneration. Cell Mol Life Sci.

[CR42] Kerur N, Hirano Y, Tarallo V, Fowler BJ, Bastos-Carvalho A, Yasuma T, Yasuma R, Kim Y, Hinton DR, Kirschning CJ, Gelfand BD, Ambati J (2013). Tlr-independent and p2x7-dependent signaling mediate Alu RNA-induced NLRP3 inflammasome activation in geographic atrophy. Invest Ophthalmol Vis Sci.

[CR43] Khan KN, Mahroo OA, Khan RS, Mohamed MD, McKibbin M, Bird A, Michaelides M, Tufail A, Moore AT (2016). Differentiating drusen: drusen and drusen-like appearances associated with ageing, age-related macular degeneration, inherited eye disease and other pathological processes. Prog Retin Eye Res.

[CR44] Kim JY, Paton JC, Briles DE, Rhee DK, Pyo S (2015). Streptococcus pneumoniae induces pyroptosis through the regulation of autophagy in murine microglia. Oncotarget.

[CR45] Kim Y, Tarallo V, Kerur N, Yasuma T, Gelfand BD, Bastos-Carvalho A, Hirano Y, Yasuma R, Mizutani T, Fowler BJ, Li S, Kaneko H, Bogdanovich S, Ambati BK, Hinton DR, Hauswirth WW, Hakem R, Wright C, Ambati J (2014). Dicer1/Alu RNA dysmetabolism induces caspase-8-mediated cell death in age-related macular degeneration. Proc Natl Acad Sci U S A.

[CR46] Klein R, Klein BE, Knudtson MD, Meuer SM, Swift M, Gangnon RE (2007). Fifteen-year cumulative incidence of age-related macular degeneration: the beaver dam eye study. Ophthalmology.

[CR47] Kole AJ, Annis RP, Deshmukh M (2013). Mature neurons: equipped for survival. Cell Death Dis.

[CR48] Kozlowski MR (2012). RPE cell senescence: a key contributor to age-related macular degeneration. Med Hypotheses.

[CR49] Kozlowski MR (2015). The arpe-19 cell line: mortality status and utility in macular degeneration research. Curr Eye Res.

[CR50] Kroemer G, Galluzzi L, Brenner C (2007). Mitochondrial membrane permeabilization in cell death. Physiol Rev.

[CR51] Kurihara Y, Kanki T, Aoki Y, Hirota Y, Saigusa T, Uchiumi T, Kang D (2012). Mitophagy plays an essential role in reducing mitochondrial production of reactive oxygen species and mutation of mitochondrial DNA by maintaining mitochondrial quantity and quality in yeast. J Biol Chem.

[CR52] Lambert NG, ElShelmani H, Singh MK, Mansergh FC, Wride MA, Padilla M, Keegan D, Hogg RE, Ambati BK. Risk factors and biomarkers of age-related macular degeneration. Prog Retin Eye Res. 2016;54:64–102. 10.1016/j.preteyeres.2016.04.003 PMC4992630 27156982

[CR53] Levonen AL, Hill BG, Kansanen E, Zhang J, Darley-Usmar VM (2014). Redox regulation of antioxidants, autophagy, and the response to stress: implications for electrophile therapeutics. Free Radic Biol Med.

[CR54] Li YJ, Jiang Q, Cao GF, Yao J, Yan B (2015). Repertoires of autophagy in the pathogenesis of ocular diseases. Cell Physiol Biochem.

[CR55] Linares JF, Duran A, Yajima T, Pasparakis M, Moscat J, Diaz-Meco MT (2013). K63 polyubiquitination and activation of mTOR by the p62-traf6 complex in nutrient-activated cells. Mol Cell.

[CR56] Liu J, Copland DA, Theodoropoulou S, Chiu HA, Barba MD, Mak KW, Mack M, Nicholson LB, Dick AD (2016). Impairing autophagy in retinal pigment epithelium leads to inflammasome activation and enhanced macrophage-mediated angiogenesis. Sci Rep..

[CR57] Lyzogubov VV, Bora PS, Wu X, Horn LE, de Roque R, Rudolf XV, Atkinson JP, Bora NS. The complement regulatory protein cd46 deficient mouse spontaneously develops dry-type age-related macular degeneration-like phenotype. Am J Pathol. 2016;186:2088–104. 10.1016/j.ajpath.2016.03.021 PMC4973660 27295359

[CR58] Mitter SK, Rao HV, Qi X, Cai J, Sugrue A, Dunn WA, Grant MB, Boulton ME (2012). Autophagy in the retina: a potential role in age-related macular degeneration. Adv Exp Med Biol.

[CR59] Mitter SK, Song C, Qi X, Mao H, Rao H, Akin D, Lewin A, Grant M, Dunn W, Ding J, Bowes Rickman C, Boulton M (2014). Dysregulated autophagy in the RPE is associated with increased susceptibility to oxidative stress and AMD. Autophagy.

[CR60] Mizumura K, Choi AM, Ryter SW (2014). Emerging role of selective autophagy in human diseases. Front Pharmacol.

[CR61] Mizumura K, Cloonan SM, Nakahira K, Bhashyam AR, Cervo M, Kitada T, Glass K, Owen CA, Mahmood A, Washko GR, Hashimoto S, Ryter SW, Choi AM (2014). Mitophagy-dependent necroptosis contributes to the pathogenesis of COPD. J Clin Invest.

[CR62] Mizushima N, Komatsu M (2011). Autophagy: renovation of cells and tissues. Cell.

[CR63] Mizushima N, Yoshimori T, Ohsumi Y (2011). The role of Atg proteins in autophagosome formation. Annu Rev Cell Dev Biol.

[CR64] Murakami Y, Matsumoto H, Roh M, Giani A, Kataoka K, Morizane Y, Kayama M, Thanos A, Nakatake S, Notomi S, Hisatomi T, Ikeda Y, Ishibashi T, Connor KM, Miller JW, Vavvas DG (2014). Programmed necrosis, not apoptosis, is a key mediator of cell loss and DAMP-mediated inflammation in dsRNA-induced retinal degeneration. Cell Death Differ.

[CR65] Nakahara T, Mori A, Kurauchi Y, Sakamoto K, Ishii K (2013). Neurovascular interactions in the retina: physiological and pathological roles. J Pharmacol Sci.

[CR66] Nakahira K, Haspel JA, Rathinam VA, Lee SJ, Dolinay T, Lam HC, Englert JA, Rabinovitch M, Cernadas M, Kim HP, Fitzgerald KA, Ryter SW, Choi AM (2011). Autophagy proteins regulate innate immune responses by inhibiting the release of mitochondrial DNA mediated by the NALP3 inflammasome. Nat Immunol.

[CR67] Nekova TS, Kneitz S, Einsele H, Stuhler G (2014). Silencing of Dicer1 temporally separates pro- and anti-apoptotic signaling and confers susceptibility to chemotherapy in p53 mutated cells. Cell Cycle.

[CR68] Nikoletopoulou V, Markaki M, Palikaras K, Tavernarakis N (2013). Crosstalk between apoptosis, necrosis and autophagy. Biochim Biophys Acta.

[CR69] Pajares M, Jimenez-Moreno N, Garcia-Yague AJ, Escoll M, de Ceballos ML, Van Leuven F, Rabano A, Yamamoto M, Rojo AI, Cuadrado A (2016). Transcription factor NFE2L2/NRF2 is a regulator of macroautophagy genes. Autophagy.

[CR70] Park JS, Kang DH, Lee DH, Bae SH (2015). Pf-4708671, a specific inhibitor of p70 ribosomal s6 kinase 1, activates nrf2 by promoting p62-dependent autophagic degradation of keap1. Biochem Biophys Res Commun.

[CR71] Reibaldi M, Longo A, Pulvirenti A, Avitabile T, Russo A, Cillino S, Mariotti C, Casuccio A. Geo-epidemiology of age-related macular degeneration: new clues into the pathogenesis. Am J Ophthalmol. 2016;161:78–93. 10.1016/j.ajo.2015.09.031 26432929

[CR72] Rodrigues GA, Maurier-Mahe F, Shurland DL, McLaughlin A, Luhrs K, Throo E, Delalonde-Delaunay L, Pallares D, Schweighoffer F, Donello J (2011). Differential effects of PPARgamma ligands on oxidative stress-induced death of retinal pigmented epithelial cells. Invest Ophthalmol Vis Sci.

[CR73] Rodriguez-Muela N, Koga H, Garcia-Ledo L, de la Villa P, de la Rosa EJ, Cuervo AM, Boya P (2013). Balance between autophagic pathways preserves retinal homeostasis. Aging Cell.

[CR74] Sarkar S (2013). Regulation of autophagy by mtor-dependent and mtor-independent pathways: autophagy dysfunction in neurodegenerative diseases and therapeutic application of autophagy enhancers. Biochem Soc Trans.

[CR75] Sato M, Sato K (2013). Dynamic regulation of autophagy and endocytosis for cell remodeling during early development. Traffic.

[CR76] Sato M, Sato K (2013). Maternal inheritance of mitochondrial DNA by diverse mechanisms to eliminate paternal mitochondrial DNA. Biochim Biophys Acta.

[CR77] Scherz-Shouval R, Elazar Z (2007). ROS, mitochondria and the regulation of autophagy. Trends Cell Biol.

[CR78] Scherz-Shouval R, Shvets E, Elazar Z (2007). Oxidation as a post-translational modification that regulates autophagy. Autophagy.

[CR79] Scherz-Shouval R, Shvets E, Fass E, Shorer H, Gil L, Elazar Z (2007). Reactive oxygen species are essential for autophagy and specifically regulate the activity of atg4. EMBO J.

[CR80] Sharma S, Helchowski CM, Canman CE (2013). The roles of DNA polymerase zeta and the y family DNA polymerases in promoting or preventing genome instability. Mutat Res.

[CR81] Shimizu S, Konishi A, Nishida Y, Mizuta T, Nishina H, Yamamoto A, Tsujimoto Y (2010). Involvement of JNK in the regulation of autophagic cell death. Oncogene.

[CR82] Singh K, Sharma A, Mir MC, Drazba JA, Heston WD, Magi-Galluzzi C, Hansel D, Rubin BP, Klein EA, Almasan A (2014). Autophagic flux determines cell death and survival in response to apo2l/trail (dulanermin). Mol Cancer.

[CR83] Sinnett D, Richer C, Deragon JM, Labuda D (1991). Alu RNA secondary structure consists of two independent 7 SL RNA-like folding units. J Biol Chem.

[CR84] Synowiec E, Blasiak J, Zaras M, Szaflik J, Szaflik JP (2012). Association between polymorphisms of the DNA base excision repair genes MUTYH and hOGG1 and age-related macular degeneration. Exp Eye Res.

[CR85] Tan PL, Bowes Rickman C, Katsanis N (2016). AMD and the alternative complement pathway: genetics and functional implications. Hum Genomics.

[CR86] Tan S, Shi H, Ba M, Lin S, Tang H, Zeng X, Zhang X (2016). Mir-409-3p sensitizes colon cancer cells to oxaliplatin by inhibiting beclin-1-mediated autophagy. Int J Mol Med.

[CR87] Taniguchi K, Yamachika S, He F, Karin M (2016). P62/sqstm1-Dr. Jekyll and Mr. Hyde that prevents oxidative stress but promotes liver cancer. FEBS Lett.

[CR88] Tarallo V, Hirano Y, Gelfand BD, Dridi S, Kerur N, Kim Y, Cho WG, Kaneko H, Fowler BJ, Bogdanovich S, Albuquerque RJ, Hauswirth WW, Chiodo VA, Kugel JF, Goodrich JA, Ponicsan SL, Chaudhuri G, Murphy MP, Dunaief JL, Ambati BK, Ogura Y, Yoo JW, Lee DK, Provost P, Hinton DR, Nunez G, Baffi JZ, Kleinman ME, Ambati J (2012). Dicer1 loss and alu rna induce age-related macular degeneration via the nlrp3 inflammasome and myd88. Cell.

[CR89] Tokarz P, Kaarniranta K, Blasiak J (2016). Inhibition of DNA methyltransferase or histone deacetylase protects retinal pigment epithelial cells from DNA damage induced by oxidative stress by the stimulation of antioxidant enzymes. Eur J Pharmacol.

[CR90] Tsao YP, Ho TC, Chen SL, Cheng HC (2006). Pigment epithelium-derived factor inhibits oxidative stress-induced cell death by activation of extracellular signal-regulated kinases in cultured retinal pigment epithelial cells. Life Sci.

[CR91] Tseng WA, Thein T, Kinnunen K, Lashkari K, Gregory MS, D'Amore PA, Ksander BR (2013). Nlrp3 inflammasome activation in retinal pigment epithelial cells by lysosomal destabilization: implications for age-related macular degeneration. Invest Ophthalmol Vis Sci.

[CR92] Tsujimoto Y, Shimizu S (2005). Another way to die: autophagic programmed cell death. Cell Death Differ.

[CR93] Tsukamoto S, Kuma A, Mizushima N (2008). The role of autophagy during the oocyte-to-embryo transition. Autophagy.

[CR94] Tsukamoto S, Kuma A, Murakami M, Kishi C, Yamamoto A, Mizushima N (2008). Autophagy is essential for preimplantation development of mouse embryos. Science.

[CR95] Ueno T, Ezaki J, Kominami E (2012). Metabolic contribution of hepatic autophagic proteolysis: old wine in new bottles. Biochim Biophys Acta.

[CR96] Vabulas RM, Hartl FU (2005). Protein synthesis upon acute nutrient restriction relies on proteasome function. Science.

[CR97] Vandenabeele P, Galluzzi L, Vanden Berghe T, Kroemer G (2010). Molecular mechanisms of necroptosis: an ordered cellular explosion. Nat Rev Mol Cell Biol.

[CR98] Viiri J, Amadio M, Marchesi N, Hyttinen JM, Kivinen N, Sironen R, Rilla K, Akhtar S, Provenzani A, D'Agostino VG, Govoni S, Pascale A, Agostini H, Petrovski G, Salminen A, Kaarniranta K (2013). Autophagy activation clears elavl1/hur-mediated accumulation of sqstm1/p62 during proteasomal inhibition in human retinal pigment epithelial cells. PLoS One.

[CR99] Wang L, Cano M, Handa JT (2014). P62 provides dual cytoprotection against oxidative stress in the retinal pigment epithelium. Biochim Biophys Acta.

[CR100] Wang L, Ebrahimi KB, Chyn M, Cano M, Handa JT (2016). Biology of p62/sequestosome-1 in age-related macular degeneration (AMD). Adv Exp Med Biol.

[CR101] Wang Y, Shen D, Wang VM, Yu CR, Wang RX, Tuo J, Chan CC (2012). Enhanced apoptosis in retinal pigment epithelium under inflammatory stimuli and oxidative stress. Apoptosis.

[CR102] Wang Y, Hanus JW, Abu-Asab MS, Shen D, Ogilvy A, Ou J, Chu XK, Shi G, Li W, Wang S, Chan CC. Nlrp3 upregulation in retinal pigment epithelium in age-related macular degeneration. Int J Mol Sci. 2016b;17:73. doi:10.3390/ijms17010073. 10.3390/ijms17010073 PMC4730317 26760997

[CR103] Yang Z, Klionsky DJ (2010). Mammalian autophagy: core molecular machinery and signaling regulation. Curr Opin Cell Biol.

[CR104] Yao J, Jia L, Khan N, Lin C, Mitter SK, Boulton ME, Dunaief JL, Klionsky DJ, Guan JL, Thompson DA, Zacks DN (2015). Deletion of autophagy inducer rb1cc1 results in degeneration of the retinal pigment epithelium. Autophagy.

[CR105] Ye F, Kaneko H, Hayashi Y, Takayama K, Hwang SJ, Nishizawa Y, Kimoto R, Nagasaka Y, Tsunekawa T, Matsuura T, Yasukawa T, Kondo T, Terasaki H (2016). Malondialdehyde induces autophagy dysfunction and VEGF secretion in the retinal pigment epithelium in age-related macular degeneration. Free Radic Biol Med.

[CR106] Yonekawa T, Thorburn A (2013). Autophagy and cell death. Essays Biochem.

[CR107] Zatloukal K, Stumptner C, Fuchsbichler A, Heid H, Schnoelzer M, Kenner L, Kleinert R, Prinz M, Aguzzi A, Denk H (2002). P62 is a common component of cytoplasmic inclusions in protein aggregation diseases. Am J Pathol.

[CR108] Zhan J, He J, Zhou Y, Wu M, Liu Y, Shang F, Zhang X. Crosstalk between the autophagy-lysosome pathway and the ubiquitin-proteasome pathway in retinal pigment epithelial cells. Curr Mol Med. 2016;16:487–95. 10.2174/1566524016666160429121606 27132793

[CR109] Zhang C, Yang L, Wang XB, Wang JS, Geng YD, Yang CS, Kong LY (2013). Calyxin Y induces hydrogen peroxide-dependent autophagy and apoptosis via JNK activation in human non-small cell lung cancer NCI-H460 cells. Cancer Lett.

[CR110] Zhang J, Bai Y, Huang L, Qi Y, Zhang Q, Li S, Wu Y, Li X (2015). Protective effect of autophagy on human retinal pigment epithelial cells against lipofuscin fluorophore a2e: implications for age-related macular degeneration. Cell Death Dis.

[CR111] Zhang L, Xie T, Tian M, Li J, Song S, Ouyang L, Liu B, Cai H (2016). GAMDB: a web resource to connect microRNAs with autophagy in gerontology. Cell Prolif.

[CR112] Zhou Q, Li H, Xue D (2011). Elimination of paternal mitochondria through the lysosomal degradation pathway in C. elegans. Cell Res.

[CR113] Zhou R, Yazdi AS, Menu P, Tschopp J (2011). A role for mitochondria in NLRP3 inflammasome activation. Nature.

[CR114] Zhu Y, Zhao KK, Tong Y, Zhou YL, Wang YX, Zhao PQ, Wang ZY (2016). Exogenous NAD(+) decreases oxidative stress and protects H2O2-treated RPE cells against necrotic death through the up-regulation of autophagy. Sci Rep.

